# HRAS overexpression predicts response to Lenvatinib treatment in gastroenteropancreatic neuroendocrine tumors

**DOI:** 10.3389/fendo.2022.1045038

**Published:** 2023-01-20

**Authors:** Chiara Liverani, Chiara Spadazzi, Toni Ibrahim, Federica Pieri, Flavia Foca, Chiara Calabrese, Alessandro De Vita, Giacomo Miserocchi, Claudia Cocchi, Silvia Vanni, Giorgio Ercolani, Davide Cavaliere, Nicoletta Ranallo, Elisa Chiadini, Giovanna Prisinzano, Stefano Severi, Maddalena Sansovini, Giovanni Martinelli, Alberto Bongiovanni, Laura Mercatali

**Affiliations:** ^1^ Bioscience Laboratory, IRCCS Istituto Romagnolo per lo Studio dei Tumori (IRST) “Dino Amadori”, Meldola, Italy; ^2^ Osteoncology, Bone and Soft Tissue Sarcomas and Innovative Therapies Unit, IRCCS Istituto Ortopedico Rizzoli, Bologna, Italy; ^3^ Pathology Unit, “Morgagni-Pierantoni” Hospital, Forlì, Italy; ^4^ Unit of Biostatistics and Clinical Trials, IRCCS Istituto Romagnolo per lo Studio dei Tumori (IRST) “Dino Amadori”, Meldola, Italy; ^5^ General and Oncologic Surgery, “Morgagni-Pierantoni” Hospital, Forlì, Italy; ^6^ Osteoncology and Rare Tumors Center, IRCCS Istituto Romagnolo per lo Studio dei Tumori (IRST) “Dino Amadori”, Meldola, Italy; ^7^ Unit of Nuclear Medicine, IRCCS Istituto Romagnolo per lo Studio dei Tumori (IRST) “Dino Amadori”, Meldola, Italy; ^8^ Scientific Directorate, IRCCS Istituto Romagnolo per lo Studio dei Tumori (IRST) “Dino Amadori”, Meldola, Italy

**Keywords:** nen, primary cultures, Lenvatinib efficacy, HRAS overexpression, predictive marker

## Abstract

**Introduction:**

Neuroendocrine neoplasms (NENs) are a rare group of tumors exceptionally heterogeneous, with clinical presentation ranging from well differentiated more indolent tumors to poorly differentiated very aggressive forms. Both are often diagnosed after the metastatic spread and require appropriate medical treatment. A high priority need in the management of this disease is the identification of effective therapeutic strategies for advanced and metastatic patients. The recent TALENT trial demonstrated the efficacy of lenvatinib, a multi-tyrosine kinase inhibitor, in patients with gastroenteropancreatic neuroendocrine tumors (GEP-NETs) with no other treatment indication. Further development of this drug in advanced NETs is warranted.

**Methods:**

We investigated potential clinical and molecular determinants of lenvatinib response in human primary cultures derived from patients with GEP-NET of different grades and sites of origin. We correlated response to treatment with patient clinical characteristics, with the mutational status of 161-cancer associated genes and with the expression levels of MKI-related genes.

**Results:**

Lenvatinib exerted a significant antitumor activity in primary GEP-NET cells, with median survival inhibitions similar or higher than those of standard frontline treatments. Of the 11 primary cultures analyzed in our case series, 6 were classified as responder showing a significant survival inhibition, and 5 as non-responder. We observed that the overexpression of HRAS in the original tumor tissue compared to the matched healthy tissue significantly correlated with responsiveness of primary cells to lenvatinib (p=.048). All 5 non-responder cultures showed normal HRAS expression, while of the 6 responder cultures, 4 had HRAS overexpression. Overexpression of HRAS was not associated with gene mutation. None of the other evaluated clinical variables (grade, Ki67, site of origin and syndromic disease) or molecular markers correlated with response.

**Discussion:**

Lenvatinib appears to be a highly effective drug for the treatment of NETs. The evaluation of HRAS expression in the tumor tissue might improve patient selection and optimize therapeutic outcome.

## 1 Introduction

Neuroendocrine neoplams (NENs) are a rare group of tumors that arise in various anatomic locations (1). The most common sites of origin are the gastro-enteropancreatic (GEP) tract and the lung. NENs are classified according to their sites of origin. Gastroenteropancreatic (GEP)-NENs are divided into grade (G) 1 and G2, G3 neuroendocrine tumors (NETs) that have well-differentiated morphology and Ki-67 ≤2% for G1, 3-20% for G2 and >20% for G3, and neuroendocrine carcinomas (NECs) with poorly differentiated morphology and Ki-67 >20% (2, 3). The estimated annual incidence is 6.9 cases per 100,000 person-year and has increased more than 6-fold over the last 4 decades (4, 5). The prevalence of NETs is currently over 170,000 patients only in the United States and will continue to grow (6, 7). The disease is exceptionally heterogeneous, with clinical presentation ranging from well differentiated more indolent tumors to poorly differentiated very aggressive forms. Both are often diagnosed after the metastatic spread and require appropriate medical treatment (8). NENs are therefore a great public health problem. Unfortunately, few oncogenic mutations are known, limiting the availability of candidate targets for therapeutic intervention and biomarkers for patient stratification (9). Few drugs have been introduced in clinical practice and therapeutic options for systemic intervention are limited. Surgery is the best approach in patients with resectable tumor, while somatostatin analogues, peptide receptor radionuclide therapy (PRRT) and molecular targeted drugs such as sunitinib and everolimus are indicated in patients with advanced disease (10–13). Chemotherapy, in particular temozolomide and capecitabine, have shown to be effective in some subsets of patients with inoperable or metastatic GEP-NEN (14, 15).

Recently, Capdevila et al. reported the TALENT trial demonstrating the efficacy of lenvatinib in the treatment of advanced well differentiated GEP-NETs (16). Lenvatinib is a multi-tyrosine kinase inhibitor (MKI) targeting vascular endothelial growth factor receptors (VEGFR) 1-3, fibroblast growth factor receptors (FGFR) 1-4, platelet-derived growth factor receptor (PDGFR) α and the proto-oncogenes RET and KIT (17, 18). Lenvatinib represents a novel therapeutic opportunity for GEP-NET patients progressing from targeted therapies or somatostatin analogues with no other treatment indication (16). In the TALENT study, despite most of the patients requiring one or more dose reduction, the overall response rate (ORR) assessed centrally was 44.2% in patients with pancreatic NET and 16.4% in patients with gastrointestinal NET. This ORR is of note especially for patients with high and symptomatic tumor burden and it is the highest reported in a clinical trial with MKI (19, 20). However, the treatment approach of NEN patients lacks clinically validated tissue or blood biomarkers to identify patients who are likely to benefit from a specific therapy, improving efficacy and avoiding unnecessary side effects. The discovery of key molecular alterations that predict therapy response has dramatically changed the success rate of several targeted compounds. As an example, tyrosine kinase inhibitors (TKIs) targeting EGFR, initially tested in an unselected population, have been of limited usefulness until the identification of EGFR gene mutations (21). They now represent the first-line therapy in the treatment of non-small cell lung cancer (NSCLC). Few studies report putative biomarkers of response to lenvatinib, but no conclusive data have been obtained. Lee et al. identified a combination of 5 serum cytokines that can predict patients with metastatic renal cell carcinoma that may benefit from second-line treatment with lenvatinib-plus-everolimus (22). Tahara et al. demonstrated that Angiopoietin 2 may be predictive of lenvatinib sensitivity in patients with thyroid cancer (23). In the era of precision medicine, evidence for a biomarker-based approach is crucially needed especially in patients with rare tumors.

The lack of reliable NEN models has represented a barrier for the identification of driver molecular alterations associated with disease pathogenesis, progression and responsiveness to anticancer agents (24). Few NEN cell lines are currently available and do not display a well-differentiated neuroendocrine phenotype (25). Moreover, the engraftment rate of NEN cells in murine models is less than 10% (26). The development of more efficient and informative preclinical models is urgently needed. To this aim, the use of patient-derived primary cultures enable the mapping of drug sensitivity and molecular profiles at individual level, representing a key technology for precision medicine (27, 28).

Here we established human primary cultures from GEP-NEN of different grades and sites of origin and assessed their sensitivity to lenvatinib in comparison with standard treatment agents for NEN patients. We characterized primary cultures to identify potential clinical and molecular markers with treatment predictive value.

## 2 Materials and methods

### 2.1 Case series

The study involved eleven patients with grade 1, grade 2 or grade 3 NETs who underwent surgical treatment at the Department of Surgical Oncology of the “Morgagni-Pierantoni” Hospital, Forlì, Italy. The protocol was approved by the Romagna Ethics Committee (CEROM) and performed according to Good Clinical Practice standards and the Declaration of Helsinki. Patients eligible for the study must have been adults (at least 18 years of age) of both sexes, undergoing surgery for NENs and must have provided written, informed consent. Included patients may have undergone or may be still in treatment, including chemotherapy (also neo-adjuvant settings), targeted therapy, radiotherapy, somatostatin analogue therapies and combination therapy.

### 2.2 Compounds

Lenvatinib mesilate (lenvatinib) were kindly provided by Eisai Co., Ltd. (Ibaraki, Japan). Everolimus was kindly provided by Novartis (NJ, USA). Temozolomide was purchased by Sigma-Aldrich (Sigma-Aldrich, Steinheim, Germany).

### 2.3 Establishment of primary cell culture

Patient-derived NEN cell cultures were isolated from surgical specimens. Prior to tissue processing, all specimens were analyzed by an expert pathologist who confirmed the presence of tumor cells in the surgical material. Tumor specimens were processed within 3 hours from resection. Samples were washed twice in sterile phosphate buffered saline (PBS) and sliced into 1-2 mm^3^ pieces with a surgical scalpel. The obtained pieces were incubated in 2 mg/ml collagenase type I (Millipore Corporation, Billerica, MA, USA) at 37°C in stirring conditions for 30 min. Then, digestion was blocked by adding Dulbecco’s modified Eagle’s medium (DMEM) supplemented with 10% fetal bovine serum, 1% glutamine and 1% penicillin/streptomycin. The solution was filtered using 100-µm sterile filters (CellTrics, Partec, Münster, Germany). Cells were counted and seeded in monolayer cultures at a density of 80,000 cells/cm^2^ and maintained in complete DMEM medium at 37°C in a 5% CO_2_ atmosphere. All the experiments were conducted using low-passage primary cultures.

### 2.4 Drug testing

25,000 cells/well were seeded in 96-well plates. Cells were allowed to recover for 72 hours before treatment. The following concentrations were used on the basis of the peak plasma concentration of each tested compound obtained from pharmacokinetic clinical data: folfox 1 ug/ml oxaliplatin plus 100 ng/ml 5-fluorouracile (29), everolimus (eve) 0.1 ug/ml (30), temozolomide (tmz) 25 μM (31) and lenvatinib (lenva) 0.6 ug/ml (32). Drug efficacy was evaluated by MTT assay. Briefly, controls and treated samples were incubated with 0.5 mg/ml of MTT solution (Sigma Aldrich) in DMEM for 2 hours at 37°C. Cell viability was determined by reading the absorbance at 550 nm. Survival percentages were calculated as the average absorbance of treated cells over the absorbance of untreated controls.

### 2.5 Quantitative real-time reverse transcriptional-PCR (qRT-PCR)

Total mRNA was isolated using TRIzol Reagent (Invitrogen) and reverse-transcribed using the iScript cDNA Synthesis Kit (BioRad, Hercules, CA, USA) with the following incubation cycles: 25°C for 5 min, 42°C for 20 min, 47°C for 20 min, 50°C for 15 min and 5 min at 85°C. Real-Time PCR was performed on the 7500 Real-Time PCR System using the SYBR Select Master Mix and the Taqman Universal Master Mix (Applied Biosystems, Foster City, CA, USA). Amplification was performed in a final volume of 20 µl containing 2x Gene expression master Mix (Applied Biosystem), 2 µl of cDNA in a total volume of 20 µl. The reaction mixtures were incubated for 2 min at 50°C, 10 min at 95°C followed by 40 PCR cycles at 95°C for 15 sec and 60°C for 1 min for overall markers. The amount of transcripts was normalized to the endogenous reference genes β-actin and HPRT and expressed as n-fold mRNA levels relative to a calibrator using a comparative threshold cycle (Ct) value method (ΔΔCt). The RNA extracted from untreated cells was used as the calibrator.

### 2.6 Focus Oncomine panel

We used an amplicon-based DNA/RNA NGS assay, known as Oncomine™ Comprehensive Assay v3 (OCAv3) (Thermo Fisher Scientific) covers 161 cancer-associated genes: 87 genes with hotspot mutations, 43 genes with focal CNV gains, 48 genes with full CDS for DEL mutations and 51 gene-fusion drivers. For all patients, 5 FFPE tumor sections of 5 µm were used for DNA and RNA extraction using the Maxwell RSC DNA and RNA FFPE Kit (Promega, Madison, WI, USA), following the manufacturer’s protocol. DNA and RNA concentrations were determined by fluorometric quantitation using a Qubit 4.0 Fluorometer with Qubit DNA dsDNA HS Assay Kit and Qubit RNA HS Assay Kit (Thermo Fisher), as appropriate. Complementary DNA (cDNA) synthesis prior to library preparation for RNA panel was carried out using SuperScript™ IV VILO™ Master Mix (Thermo Fisher Scientific). Library preparation was performed using the Oncomine™ Comprehensive Assay v3 DNA/RNA Chef-Ready panel, designed for use with the Ion Chef™, following manufacturer’s instructions, with 10 ng input DNA and RNA per sample. The libraries were loaded onto Ion Chef System (Thermo Fisher Scientific) for template preparation, using Ion 540™ Kit-Chef, and finally sequenced on the Ion S5 Plus platform (Thermo Fisher Scientific) using the Ion 540 Chips (Thermo Fisher Scientific). Primary analysis was carried out using a Torrent Suite Server™ (5.12.3) to perform initial quality control, including chip loading density, median read length and number of mapped reads. Afterwards, a second analysis was performed by Ion Reporter™ Software (5.16), hosting informatics tools for variants, filtering, and annotations. Variants were identified with VAF greater than or equal to 5% with coverage greater than 500X and clinically relevant. CNV algorithm in Ion Reporter is used with the following features: minimum % cellularity for accurate CNV calling is 50% and Median Absolute Pairwise Difference (MAPD) is <0.5. The RNA panel was able to identify 51 rearrangements. A fusion was classified as present with greater than 500,000 mapped reads, providing evidence for the fusion. The performance of the DNA/RNA panel was established, using Seraseq Lung & Brain CNV Mix, +6 copies and FFPE Fusion RNA Reference Material v4 (Seracare Life Sciences, Inc.).

### 2.7 Statistical analysis

Three independent replicates were performed for each experiment. Data are presented as mean ± standard deviation (SD) or mean ± standard error, as stated, with *n* indicating the number of replicates. Differences between groups were assessed by a two-tailed Student’s t-test and accepted as significant at *p*<.05. Correlation of clinical and molecular variable with lenvatinib responsiveness was performed by Fisher’s exact test and accepted as significant at *p*<.05.

## 3 Results

### 3.1 Descriptive characteristics

The main clinical and histological characteristics of the 11 patients analyzed in this study are shown in **Table 1**. Median follow up was 30 months (range 19-72). Nine patients (81.8%) were males and two (18.2%) were females. Median age at the time of diagnosis was 79 years (range 36-84). The site of the primary tumor was pancreas in four patients (36.4%), stomach in two patients (18.2%) and ileum in five patients (45.4%). Eight patients (72.7%) had grade (G) 1 well-differentiated NET, two patients (18.2%) had G2 well-differentiated NET and one patient (9.1%) had a G3 well-differentiated NET. Ten patients (90.9%) showed positive ^68^Gallium-positron emission tomography/computerized tomography (^68^Ga-PET/CT), while for one patient was not performed. Three patients (27.3%) showed positive ^18^F-fluorodeoxyglucose (^18^FDG)-PET/CT scan, while the ^18^F-FDG PET/CT scan for the remaining patients was not performed. Two patients (18.2%) had a syndromic disease caused by hormonal hypersecretion. Only two patients (18.2%) developed metastatic disease. Three patients (27.3%) underwent first line therapy. One patient showed stable disease after receiving 3 subcutaneous administrations of lanreotide (120 mg every 28 days). One patient was treated with sandostatine (30 mg every 28 days) showing stable disease at the 3 months re-evaluation. One patient (G3 NET) was treated with folfox4 every 2 weeks (oxaliplatin 85 mg/m^2^ and fluorouracil 400 mg/m^2^ bolus on day 1, then fluouracil 600 mg/m^2^ over 22 hours on days 1 and 2) showing a partial response after 7 cycles.

**Table 1:** Clinicopathological characteristics.

	n (%)
**Median follow up, months (range)**	30 (19-72)
**Age at diagnosis, years (range)**	79 (36-84)
Gender	
Male	9 (81.8)
Female	2 (18.2)
Site of disease	
Pancreas	4 (36.4)
Stomach	2 (18.2)
Ileum	5 (45.4)
Grading	
G1	8 (72.7)
G2	2 (18.2)
G3	1 (9.1)
^68^Ga-PET/CT Octreoscan	
Negative	0 (0.0)
Positive	10 (90.9)
nd	1 (9.1)
^18^F-FDG PET/CT	
Negative	0 (0.0)
Positive	3 (27.3)
nd	8 (72.7)
Metastatic disease	
Yes	2 (18.2)
No	9 (81.8)
Syndromic disease	
Yes	2 (18.2)
No	8 (72.7)
nd	1 (9.1)
Best response to firs line therapy	
PR	1 (9.1)
SD	2 (18.2)
nd	8 (72.7)

G, grade; ^68^Ga, Gallium-68; nd, not determined: PR partial, response; SD, stable disease.

### 3.2 Establishment of NEN primary cultures

**Figure 1:**
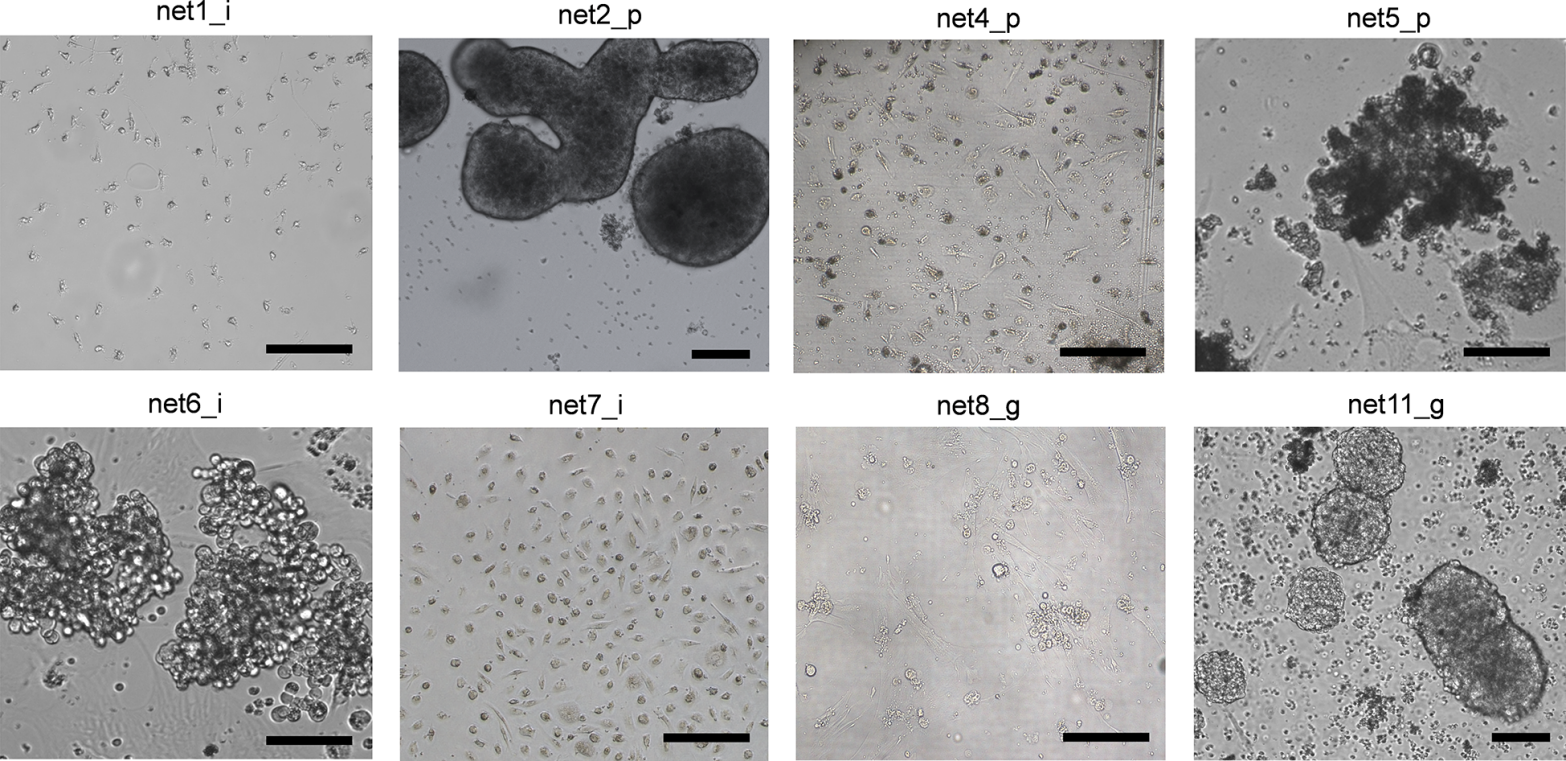
Human primary NEN cells showed varied morphology in monolayer culture. Bright field representative images of different primary NEN cells with ileal, pancreatic and gastric origins. Scale bar: 50 µm.

Primary NEN cells showed limited viable time after recovery from the tumor specimens. None of the isolated cultures was stable for more than 3 passages. For this reason, all treatments were performed within 1 week from isolation. Examples of primary NEN cell appearance are reported in **Figure 1**. All cells displayed an epithelioid morphology as confirmed by an expert pathologist. Net1 derived from an ileal G1 lesion and appeared as cells with very small dimensions with mixed shape. Net2 and Net5 derived from a G1 and a G2 pancreatic tumor, respectively. Net2 cells formed dense, large aggregates with varied morphology, some of them showing spheroid-like appearance; Net5 cells formed large disorganized aggregates. Net4 derived from a G1 pancreatic tumor and appeared as isolated cells mixed with fibroblasts. Net6 and Net7 derived from two G1 ileal tumors: Net6 formed large aggregates of rounded-shaped cells, while Net7 appeared as isolated cells mixed with fibroblasts. Net8 derived from a G3 gastric tumor and appeared as small aggregates of cells with rounded morphology over a fibroblast layer. Finally, Net11 derived from a G1 gastric tumor and appeared as small rounded cells forming aggregates with varied dimensions and spheroid-like morphology.

### 3.3 Efficacy of lenvatinib and conventional NEN therapies in primary cultures

**Figure 2:**
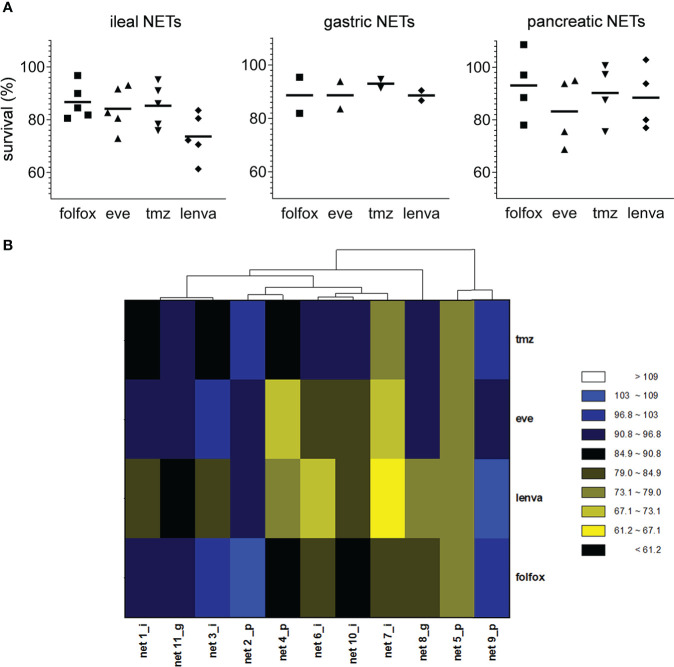
Effect of pharmacological treatments on cell viability in human primary NEN cultures. **(A)** Survival percentages values according to tumor site of origin in human primary NEN culture treated with folfox, everolimus (eve), temozolomide (tmz) and lenvatinib (lenva) compared to untreated controls. Data represent median value (line) and single values for each primary culture (*n*=1 per patient, 3 technical replicates). **(B)** Heatmap visualization comparing survival percentages for folfox, eve, tmz and lenva in human primary NEN cultures. Each column represents an individual primary culture. The dendrogram displays Pearson’s clustering distance. The color code represents the scaled survival percentages for each drug.

**Figure 3:**
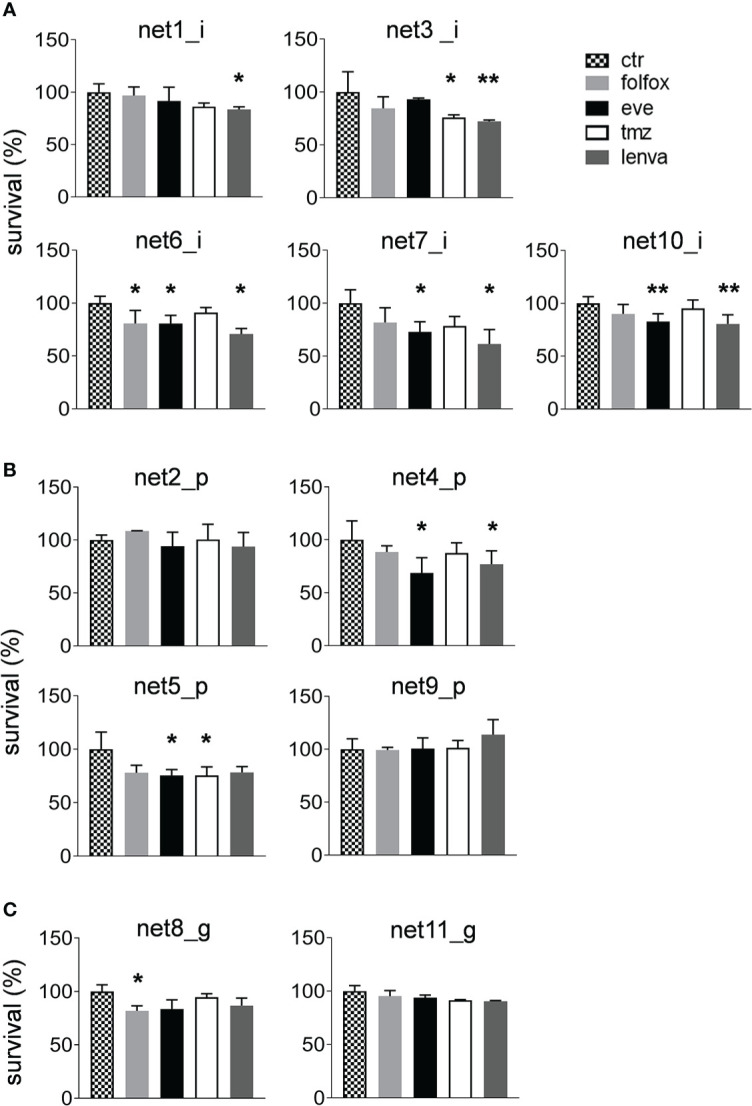
Sensitivity of primary NEN cells to standard upfront treatment or to lenvatinib. **(A)** Survival percentages of ileal primary NEN cultures (Net1, Net3, Net6, Net7, Net10) treated with folfox, eve, tmz and lenva compared to untreated controls. Data represent mean ± S.D. (*n*=3) ^*^ *p*<.05, two-tailed Student’s t-test. **(B)** Survival percentages of pancreatic primary NEN cultures (Net2, Net4, Net5, Net9) treated with folfox, eve, tmz and lenva compared to untreated controls. Data represent mean ± S.D. (*n*=3) ^*^ *p*<.05, two-tailed Student’s t-test. **(C)** Survival percentages of gastric primary NEN cultures (Net8, Net11) treated with folfox, eve, tmz and lenva compared to untreated controls. Data represent mean ± S.D. (*n*=3) ^*^ *p*<.05, ^**^ *p*<.001, two-tailed Student’s t-test.

We assessed the sensitivity of the eleven primary NEN cultures to lenvatinib. Efficacy was compared to that of fluoruracil plus oxapliplatin (folfox), temozolomide and everolimus which represent approved drugs for the treatment of NEN patients. Overall, lenvatinib exerted an antitumor activity in NET of ileal origin with a median survival inhibition of 25.6%. Also in gastric and pancreatic NET, lenvatinib showed good efficacy with a median survival inhibition of 11.0% and 11.6%, respectively (**Figure 2A**). Compared to other drugs, lenvatinib was the most effective treatment in ileal and gastric NETs, while in pancreatic NETs everolimus resulted to be the most active compound. Cluster heatmap of survival percentages in the primary cultures displayed response groups independent from the sites of origins (**Figure 2B**). Taking into consideration each primary culture, lenvatinib induced a significant inhibition of survival in all tested ileal cells (*p*=.0439 for Net1, *p*=.0050 for Net3, *p*<.001 for Net6, *p*=.0094 for Net7 and *p*<.001 for Net10) (**Figure 3A**). Folfox induced a significant inhibition of survival only in Net6 (*p*=.0297), everolimus exerted a significant activity in Net6 (*p*=.0045), Net7 (*p*=.0311) and Net 10 (*p*<.001), while temozolomide was effective only in Net3 (*p*=.0346) (**Figure 3A**). In primary pancreatic cells, lenvatinib induced a significant inhibition of survival in Net4 (*p*=.0433), while it was not significantly active in Net2, Net5 and Net9 (**Figure 3B**). Folfox was not effective in pancreatic cells, everolimus was effective in Net4 (*p*=.0187) and Net5 (*p*=.0358) but not in Net2, and Net9, temozolomide was effective only in Net5 (*p*=.0354) (**Figure 3B**). In gastric primary cells lenvatinib was not significantly effective in any of the two primary cultures (**Figure 3C**). Net11 was not sensitive to any of the other tested drugs. In Net8 cells, folfox showed significant inhibition of cell survival (*p*=.0090). Everolimus was not effective in any of the two gastric primary cultures (**Figure 3C**). It is interesting to notice that for Net8, sensitivity of tumor cells to folfox was confirmed in the clinical setting. The patient was treated in neoadjuvant setting with folfox IV regimen for 12 cycles and showed a partial response (**Table 1**). Unfortunately, no other clinical data on treatment were available to match results with those obtained in primary cells, as most of the patients did not receive any first line therapy (**Table 1**).

### 3.4 Correlation of lenvatinib efficacy with clinical and molecular characteristics

**Figure 4:**
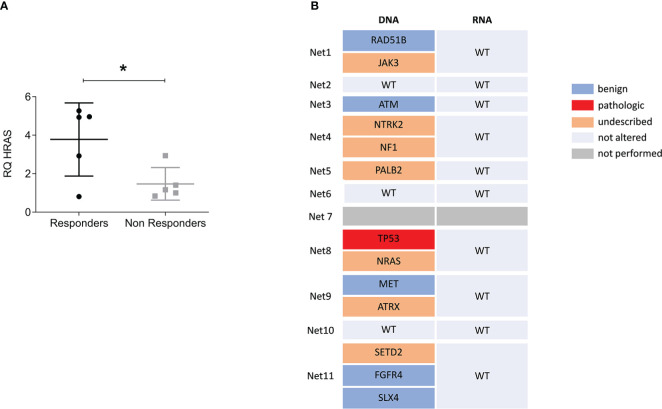
HRAS overexpression but not mutation predicts responsiveness to lenvatinib. **(A)** Relative expression levels of HRAS in tumor tissues over matched healthy tissues comparing responder versus non responder patients. Data represent mean ± S.D. (*n*=3) ^*^ *p*<.05, two-tailed Student’s t-test. **(B)** Variant data from the Oncomine Focus Assay panel identified in the NEN patient cohort. The list of genes includes only those found to be altered. Variants were classified as benign, pathologic and of unknown significance. Wild type (WT) indicate that no alteration were found. For Net7 patient the assay was not performed.

Correlation of lenvatinib efficacy with patient clinical and molecular characteristics is reported in **Table 2**. Of the eleven primary cultures analyzed in our case series, six were classified as responder with a significant survival inhibition in treated cells compared to controls, and five as non-responder. We observed that the overexpression of HRAS in the tumor tissues compared to matched healthy tissues significantly correlated with responsiveness of primary NET cells to lenvatinib (*p*=.048) (**Table 2** and **Figure 4A**). All five non-responder patients showed normal HRAS expression, while of the six responder patients, four showed HRAS overexpression and only one normal expression. For one responder patient HRAS was not determined. HRAS overexpression did not correlate with gene mutation. Tissue samples were subject to an NGS targeted sequencing assay for the detection of single nucleotide variants (SNVs), copy number variations (CNVs), gene fusions, and indels from 161 cancer driver genes. None of the analyzed primary tissues showed mutation in HRAS, and few molecular alterations were detected in the tumor tissues confirming the extremely low mutational burden of these tumors (**Figure 4B**). However, mutations in various genes related to TKs were detected in the tumor samples (JAK3, NRAS, NTRK2, NF1, SETD2) suggesting frequent alteration of TK pathways for neuroendocrine neoplasms.

**Table 2:** Correlation of response to Lenvatinib with clinical and molecular data.

	Levatinib			*p*-value
	Non-responder (%)	Responder (%)	Total	
**Overall**	5 (45.5)	6 (54.5)	11	
Syndromic disease				
yes	4 (50.0)	4 (50.0)	8^*^	.467
no	0 (0.0)	2 (100.0)	2
**Ki67**				
≤2	2 (25.5)	6 (75.5)	8	.061
>2	3 (100.0)	0 (0.0)	3
Grade				
1	2 (25.5)	6 (75.5)	8	
2	2 (100.0)	0 (0.0)	2	.061
3	1 (100.0)	0 (0.0)	1	
Site of origin				
G.I.	2 (28.6)	5 (71.4)	7	.242
pancreas	3 (75.5)	1 (25.5)	4
HRAS overexpression				
no	5 (83.3)	1 (16.7)	6^†^	*.048*
yes	0 (0.0)	4 (100.0)	4	

^*^For 1 patient data on syndromes were not available.

^†^HRAS expression was not evaluable in 1 patient.

p-value <.05, Fisher’s exact test.

Finally, none of the others analyzed markers and clinical variables (grade, Ki67, site of origin and syndromic disease) showed correlation with lenvatinib responsiveness (**Table 2** and **Supplementary Figure 1**), although this result may depend on the small sample size.

## 4 Discussion

A high priority unmet need in the management of NEN patients is the improvement of the actual therapeutic landscape for patients with metastatic or locally-advanced disease. In the recent years, important scientific advances have been introduced for the treatment of NENs. However, predictive factors to select patients that would benefit from targeted treatments and to guide sequencing of systemic regimens are lacking (33). Advances have been limited by the lack of murine and human cell line models that do not accurately represent the well-differentiated and slow proliferative phenotypes of NETs and fail to reflect the inter-patient variability (34). Lenvatinib, a potent inhibitor of VEGF receptors (VEGFR1-3) and other pro-oncogenic receptor TKs, was recently tested in patients with G1 and G2 GEP-NETs showing encouraging results. Despite this, biomarkers to optimize the outcome of MKI treatment and avoid unnecessary side effects are still to be established (35). Here we evaluated the antitumor activity of lenvatinib in eleven primary GEP-NET cultures of different grade and sites of origin investigating clinical and molecular markers with potential predictive value. We observed that lenvatinib exerts a significant inhibition of cell growth in primary GEP-NET cells. No differences were found according to tumor site of origin. Compared to standard therapeutic drugs used in frontline treatment for NEN patients, lenvatinib resulted the most active compound in ileal and gastric primary cells, while in pancreatic NETs everolimus was the most effective drug. Of note, folfox was ineffective in the majority of primary cultures, confirming the scarce sensitivity of low grade neuroendocrine tumors to chemotherapy (14). We next demonstrated that overexpression of HRAS in the patient tumor tissue compared to matched healthy tissues significantly correlates with responsiveness of primary cells to lenvatinib. Of the six responder patients, four showed HRAS overexpression in the tumor tissue. The Ras-Raf-MEK-ERK pathway, downstream of TK receptors, is a key signaling pathway involved in tumorigenesis and angiogenesis of about a third of all human cancers, including NETs (36). The prevalence of HRAS overexpression and mutation in NET patients and its correlation with clinical outcomes have not been investigated. According to our evidence, HRAS overexpression can be found in about 40% of GEP-NET tumor tissues and might portend a higher sensitivity to lenvatinib treatment. Moreover, considering that Capdevila et al. propose lenvatinib to be effective in GEP-NET patients that progressed after treatment with targeted agents (16), it would be interesting to investigate the prevalence of HRAS overexpression in patients with resistance to upfront therapies.

Overexpression of HRAS was not correlated with gene mutation. None of the analyzed tumor samples resulted mutated for HRAS. A similar result emerged in the exploratory biomarker analysis of the phase III Study of (E7080) lenvatinib in differentiated cancer of the thyroid (SELECT trial) (23). Tahara et al. demonstrated that lenvatinib PFS benefit observed in patients with thyroid cancer was consistent in all analyzed subgroups regardless of the BRAF or RAS mutational status in the tumor tissues (23). From our sequencing analysis, we also confirmed that NENs are characterized by an extremely low tumor mutational burden (37). Interestingly, the mutations found in our case series consistently involve genes associated with TK pathways. Our study include a limited number of patients, thus a confirmatory analysis in a larger case series is needed to understand if NENs are enriched for alterations in TK genes. Results might uncover driver mutations that are currently lacking in this disease, and confirm data emerged in the TALENT study that reported the highest ORR ever in a clinical trial with a MKI.

In addition, data on lenvatinib mechanism of action in neuroendocrine cells are missing. Lenvatinib has shown antitumor activity against multiple tumor types, such as hepatocellular carcinoma (38), differentiated thyroid cancer (DTC) (39), anaplastic thyroid cancer (ATC) (40), medullary thyroid cancer (MTC) (41), gastric cancer (42), thymic carcinoma (43) and other solid tumors (44). In these tumors lenvatinib exerted diverse mechanisms of action linked to the block of cell proliferation through the targeting of proto-oncogenes RET and KIT and their pathways, and the inhibition of angiogenesis (17, 45–47). The relevance of tumor angiogenesis in NENs is well established. NENs are characterized by an extremely high vascularization, even in the low-grade forms, and strong expression of pro-angiogenic factors such as VEGF-A (48). Investigation into the antiangiogenic effects of lenvatinib and other TKIs in these tumors should be of great relevance.

## 5 Conclusions

In conclusion, lenvatinib effectively inhibits survival of GEP-NEN cells. The evaluation of HRAS expression in the tumor tissue might improve patient selection and optimize therapeutic outcome. Future efforts should focus on understanding the exact mechanism of action of lenvatinib and TKIs in these tumors.

## Data availability statement

The dataset will be available upon request to the corresponding author, as they are part of genetic data obtained from analyses of patients.

## Ethics statement

The studies involving human participants were reviewed and approved by Romagna Ethics Committee (CEROM). The patients/participants provided their written informed consent to participate in this study.

## Author contributions

CL, CS, AB and LM designed the study. CL, CS, TI, FP, CCa, ADV, GMi, CCo, SV, GE, DC, NR, EC, GP, SS, MS, and GMa collected and analyzed all clinical and biological data. FF carried out the statistical analyses. CL, CS, AB and LM interpreted the data. CL and CS drafted the manuscript. LM and AB revised the manuscript for intellectual content. All authors contributed to the article and approved the submitted version.

## Glossary

**Table:**

CDS	coding sequence
CNV	copy number variation
Ct	cycle threshold
DMEM	Dulbecco’s modified Eagle’s medium
FFPE	formalin-fixed paraffin-embedded
FGFR	fibroblast growth factor
receptors	
GEP	gastro-enteropancreatic
GEP-NET	gastro-enteropancreatic neuroendocrine tumor
MAPD	median absolute pairwise difference
MKI	multi-tyrosine kinase inhibitor
NEC	neuroendocrine carcinoma
NEN	neuroendocrine neoplasm
NET	neuroendocrine tumor
NSCLC	nonsmall cell lung cancer
ORR	overall response rate
PBS	phosphate buffered saline
PDGFR	platelet-derived growth factor receptor
PRC	polymerase chain reaction
PRRT	peptide receptor radionuclide therapy
SD	standard deviation
TKI	tyrosine kinase inhibitor
VAF	variant allele frequency;
VEGFR	vascular endothelial growth factor receptors.
