# Supplementary material for: HRAS overexpression predicts response to Lenvatinib treatment in gastroenteropancreatic neuroendocrine tumors
Source: Front Endocrinol (Lausanne). 2023 Jan 20;13:1045038. doi: 10.3389/fendo.2022.1045038 (PMC9895371; doi:10.3389/fendo.2022.1045038)
Supplement: Supplementary file 1 [file DataSheet_1.pdf]

Supplementary Material

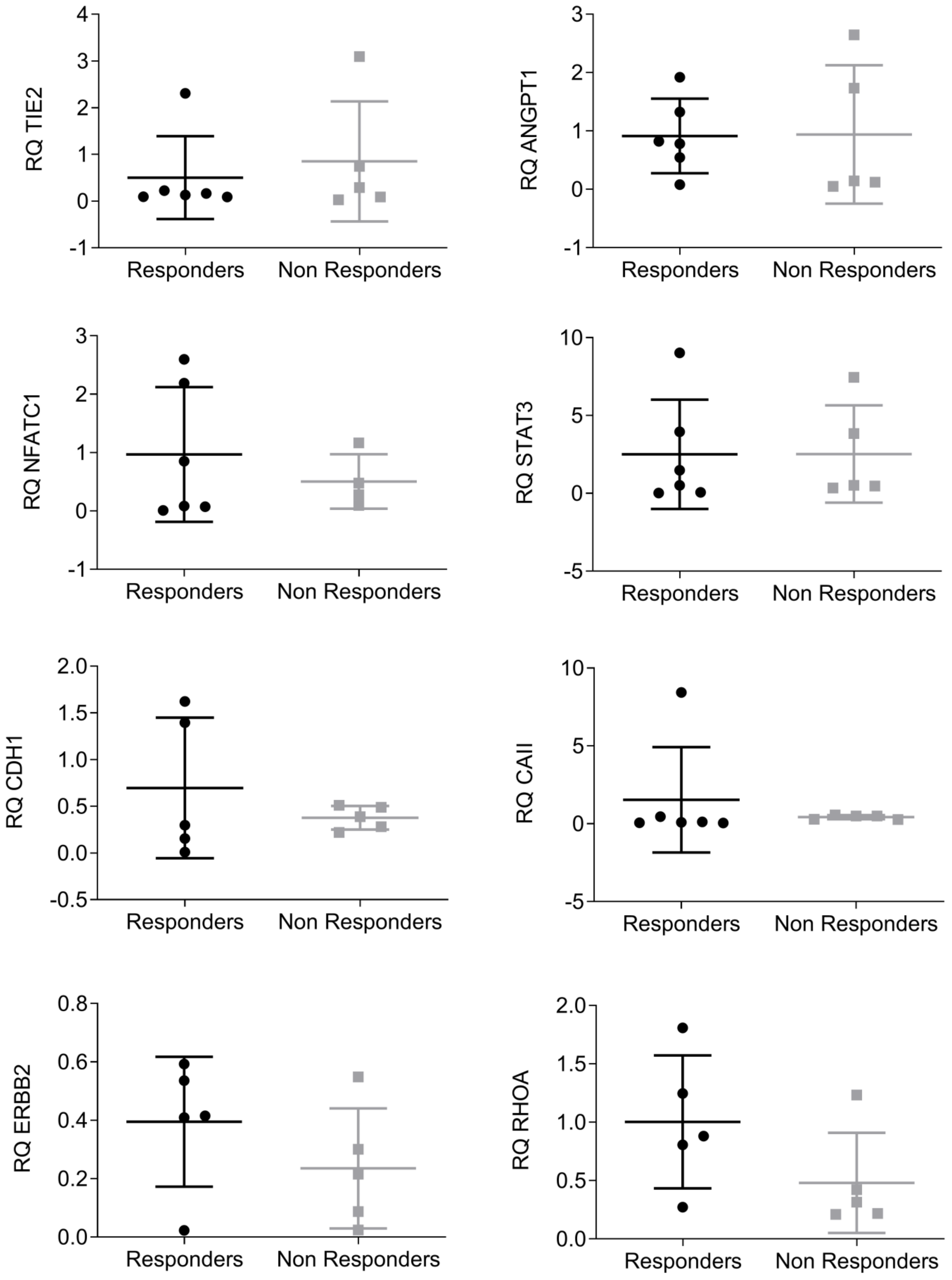

Supplementary Figure 1 Relative expression levels of TIE2, ANGPT1, NFATC1, STAT3, CDH1, CAII, ERBB2 and RHOA in tumor tissues over matched healthy tissues comparing responder versus non responder patients. Data represent mean ± S.D. (n=3) $^{*}p$<.05, two-tailed Student's t-test.
